# Depletion of adult neurogenesis exacerbates cognitive deficits in Alzheimer’s disease by compromising hippocampal inhibition

**DOI:** 10.1186/s13024-017-0207-7

**Published:** 2017-09-08

**Authors:** Carolyn Hollands, Matthew Kyle Tobin, Michael Hsu, Kianna Musaraca, Tzong-Shiue Yu, Rachana Mishra, Steven G. Kernie, Orly Lazarov

**Affiliations:** 1 Department of Anatomy and Cell Biology, College of Medicine, The University of Illinois at Chicago, 909 S Wolcott Ave, Chicago, IL 60612 USA; 2 Department of Pediatrics and Pathology & Cell Biology, Columbia University College of Physicians and Surgeons, New York, NY 10032 USA

**Keywords:** Alzheimer’s disease, Hippocampal neurogenesis, Learning and memory, Hippocampal circuit, Tau phosphorylation

## Abstract

**Background:**

The molecular mechanism underlying progressive memory loss in Alzheimer’s disease is poorly understood. Neurogenesis in the adult hippocampus is a dynamic process that continuously changes the dentate gyrus and is important for hippocampal plasticity, learning and memory. However, whether impairments in neurogenesis affect the hippocampal circuitry in a way that leads to memory deficits characteristic of Alzheimer’s disease is unknown. Controversial results in that regard were reported in transgenic mouse models of amyloidosis.

**Methods:**

Here, we conditionally ablated adult neurogenesis in APPswe/PS1ΔE9 mice by crossing these with mice expressing nestin-driven thymidine kinase (δ-HSV-TK).

**Results:**

These animals show impairment in performance in contextual conditioning and pattern separation tasks following depletion of neurogenesis. Importantly, these deficits were not observed in age-matched APPswe/PS1ΔE9 or δ-HSV-TK mice alone. Furthermore, we show that cognitive deficits were accompanied by the upregulation of hyperphosphorylated tau in the hippocampus and in immature neurons specifically. Interestingly, we observed upregulation of the immediate early gene Zif268 (Egr-1) in the dentate gyrus, CA1 and CA3 regions of the hippocampus following learning in the neurogenesis-depleted δ-HSV-TK mice. This may suggest overactivation of hippocampal neurons in these areas following depletion of neurogenesis.

**Conclusions:**

These results imply that neurogenesis plays an important role in the regulation of inhibitory circuitry of the hippocampus. This study suggests that deficits in adult neurogenesis may contribute to cognitive impairments, tau hyperphosphorylation in new neurons and compromised hippocampal circuitry in Alzheimer’s disease.

**Electronic supplementary material:**

The online version of this article (10.1186/s13024-017-0207-7) contains supplementary material, which is available to authorized users.

## Background

The mechanism underlying cognitive deficits in Alzheimer’s disease (AD) is not fully elucidated [1]. Normal age-related memory loss is thought to begin in the dentate gyrus (DG) [2]. This observation is supported by high-resolution fMRI [3–5] and cognitive studies [6–9]. In AD, neuronal loss in the entorhinal cortex is preceded by a long period of deficits in the connectivity of the hippocampal formation [10]. In the hippocampus there is an age-dependent decrease in the number of new neurons that are being continuously added to the dentate gyrus in rodents [11–16]. Similarly there is a decline in adult neurogenesis in humans [17–20]. This is compounded by changes in synaptic structure. In both rodents and humans the density of synaptic contacts formed onto granule cells of the DG is reduced with age [21, 22].

In familial Alzheimer’s disease (FAD)-linked APPswe/PS1ΔE9 mice, hippocampal neurogenesis is impaired in young adults, prior to cognitive deficits or the appearance of amyloid plaque pathology [23]. Similar observations of neurogenic impairments were independently confirmed in the 3xTg-AD mouse [24, 25] (review [26, 27]). Additionally, in vitro studies of neural progenitor cells (NPCs), isolated from the subgranular layer of APPswe/PS1ΔE9 mice, show both expression of hyperphosphorylated tau and reduced proliferation [23]. Nevertheless, there have been contradicting reports about the fate of neurogenesis in FAD, and the contribution of neurogenesis to AD remains controversial [1, 28, 29].

Significantly, there is overlap between FAD related proteins and proteins regulating adult neurogenesis (review [26, 27]). In particular, presenilin 1 (PS1) regulates neural progenitor cell differentiation in vitro and in vivo [30]. Additionally, soluble amyloid precursor protein alpha (sAPPα), a cleavage product of the amyloid precursor protein (APP), acts as a proliferation factor on NPCs in the adult brain [31, 32]. PS1/γ-secretase regulates the metabolism of critical players in neurogenesis, such as notch-1, EGF, β-catenin and cAMP response element binding protein (CREB), all of which are important neurogenic signals. Changes in PS1 function could alter these signaling factors and thus affect neurogenesis. Importantly, we have shown that downregulation of PS1 compromises the maturation of new neurons, suggesting that defective neurons incorporate into the dentate gyrus [33].

In light of that, in this study we attempted to address three fundamental questions: 1. Does depletion of hippocampal neurogenesis promote cognitive decline observed in AD? 2. Does depletion of hippocampal neurogenesis promote AD-related neuropathology? and 3. Does depletion of hippocampal neurogenesis alter the hippocampal circuit? To address these questions, we conditionally ablated neurogenesis in APPswe/PS1ΔE9 mice using nestin-regulated expression of thymidine kinase (δ-HSV-TK). We show that depletion of adult neurogenesis in APPswe/PS1ΔE9 impaired performance in contextual conditioning and pattern separation tasks. These deficits were not observed in age-matched APPswe/PS1ΔE9 mice. Furthermore, depletion of neurogenesis induced hyperphosphorylation of tau in the hippocampus. However, we observed no effect of neurogenesis on level of oligomeric Aβ in the entorhinal cortex of these mice. Importantly, we show overactivation of neurons in the dentate gyrus, CA3 and CA1 regions of the hippocampus following depletion of neurogenesis, suggesting that neurogenesis plays an important role in regulating neuronal activation in the hippocampus. In summary, this is the first study to examine the effect of reduced neurogenesis on the development of cognitive deficits and AD. Combined, the results of this study suggest that reduced levels of hippocampal neurogenesis can induce cognitive dysfunction and tau pathology characterizing AD, and interfere with hippocampal function.

## Methods

### Animals

Animal care procedures were conducted according to the National Institutes of Health Guide for the Care and Use of Laboratory Animals. Our colony is maintained via group housing (≤5 mice per cage) in a high barrier facility under a 14:10 light:dark cycle with free access to food and water. FAD-linked APPswe/PS1ΔE9 mice [34] and Nestin-δ-HSV-TK mice [35] were generated as previously described. To maintain consistency of pathology progression and avoid gender-related disparate observations, mice used in this study were all females. Mouse euthanasia was performed using isofluorane and cervical dislocation.

### Valganciclovir treatment

Valganciclovir powder was mixed into mouse chow (.09%, Valcyte, valganciclovir hydrochloride) and given ad libitum for an average dose of ~90 mg/kg/day (Custom Animal Diets, LLC (Easton, PA)). Animals were fed either Valganciclovir- containing chow or standard chow (“vehicle”) right after weaning for 2 months. At the end of this period mice were subjected to behavioral tests, i.e., contextual fear conditioning and pattern separation sequentially. A separate group of mice was subjected to pattern separation followed by a single probe test, as described in Fig. 5. Following sacrifice, brains were analyzed as described below.

### Contextual fear conditioning

Performed as previously described [36]. Conditioning was conducted in two distinct contexts: context A with the shock, and context C without the shock. Both test cages (17.8 × 17.8 × 30.5 cm) were encased by isolation cubicles. Context A had two plexiglass walls, two metal walls and a stainless steel grid floor (Coulbourn Instruments). The light and fan were turned on. A mild lemon blossom scent was used, and 70% ethanol for cleaning. Context C had the light and fan turned off, the chamber door ajar, a mild anise scent, and Clorox disinfecting wipes for cleaning. The shape of the chamber was altered with a plastic circular insert, and a plastic flooring was placed on top of the stainless steel grid floor with cage bedding added. Motion was recorded by a digital video camera mounted above the test cage. On day 0 mice were tested only in context A, then for 3 consecutive days in both A and C context with an hour separation. In test cage A the mice received a single 2 s foot shock (0.75 mA at the 185th second). FreezeFrame and FreezeView software (Actimetrics) were used for recording and analyzing freezing behavior. Percentage of freezing during the first 180 s in each context for each day was computed. Discrimination ratios were calculated using the formula:

$$ \mathrm{Discrimination}\  \mathrm{Ratio}=\left({\mathrm{Freezing}}_{\mathrm{A}}\hbox{--} {\mathrm{Freezing}}_{\mathrm{C}}\right)/\left({\mathrm{Freezing}}_{\mathrm{A}}+{\mathrm{Freezing}}_{\mathrm{C}}\right). $$

### Pattern separation

Performed as previously described [33]. Conditioning was conducted in two similar contexts: the shock context A, and the similar context B. Both cages had two clear Plexiglas walls, two grey metal walls and a stainless steel grid floor (Coulbourn Instruments). In cage A the house light and fan were turned on. A mild lemon blossom scent was used. Cages were cleaned with 70% ethanol prior to mouse placement. Test cage B differed from test cage A in that the metal walls had black and white inserts, the house light and fan were turned off and the chamber door was left ajar. A mild peppermint scent was used, and Clorox disinfecting wipes. On day 0 mice were exposed only to the training context A. For the next nine consecutive days mice were exposed to A and B in a randomized order. Discrimination ratios were calculated using the formula:

$$ \mathrm{Discrimination}\  \mathrm{Ratio}=\left({\mathrm{Freezing}}_{\mathrm{A}}\hbox{--} {\mathrm{Freezing}}_{\mathrm{B}}\right)/\left({\mathrm{Freezing}}_{\mathrm{A}}+{\mathrm{Freezing}}_{\mathrm{B}}\right). $$

### BrdU injections

Two doses of 5′-bromo-2′-deoxyuridine (BrdU; Sigma) were administered four hours apart intraperitoneally (100 mg/kg), in physiological saline. Animals were sacrificed four weeks later.

### Brain tissue processing

For immunohistochemical staining, all mice were anesthetized using overdose of isofluorane and transcardially perfused with ice-cold PBS. Removed brains were halved on the sagittal plane, and half placed into 4% paraformaldehyde and half saved for western blot.

### Immunohistochemistry

50 μm sagittal sections cut using a sliding freezing microtome (Leica Biosystems, Buffalo Grove, IL) were stored in cryoprotectant (glycerol, ethylene glycol, 1X PBS) at −20 °C. The following antibodies were used: rat anti-BrdU (1:400; Accurate Chemical & Scientific Corp., Westbury, NY), mouse anti-nestin (1:100; Millipore Corporation, Billerica, MA), goat anti-doublecortin (DCX; 1:400; Santa Cruz Biotechnology, Santa Cruz, CA), mouse anti-NeuN (1:400, Millipore, Temecula, CA), rabbit anti-Egr-1 (1:250, Santa Cruz Biotechnology, Santa Cruz, CA), goat anti-green fluorescent protein (GFP, 1:1000, Abcam, Inc. Cambridge, MA) and mouse anti-green fluorescent protein (GFP, 1:200; Santa Cruz Biotechnology, Santa Cruz, CA). Secondary antibodies from Jackson ImmunoResearch Laboratories (West Grove, PA): biotinylated species-specific anti-IgG (all used at 1:250), Cy3-conjugated Donkey anti-Rabbit (1:250), Cy3-conjugated Donkey anti-Rat (1:250), Alexa Fluor 647 (AF647)-conjugated Donkey anti-Mouse and anti-Rat (1:250), Cy2-conjugated Streptavidin (1:250), DAPI Nucleic Acid Stain (1:2500, Life Technologies, Grand Island, NY).

### Stereological quantification

Cell counts were performed using design-based stereology (StereoInvestigator version 8, MBF Bioscience). For the analysis, every sixth section of brain tissue was quantified by applying the Nv × VRef method. Sections were traced using a Zeiss AX10 microscope (Carl Zeiss Ltd., Hertfordshire, England) in low magnification (5×) and counting was performed at high magnification (63×), counting frame = 100 μm × 100 μm, grid size 100 μm × 100 μm, and all sections were counted using 12.5-μm top and bottom guard zones.

### Dot-blot for Oligomeric Aβ

Entorhinal cortex underwent extraction for isolating the water soluble Aβ fraction for dot-blot analysis as previously described [37]. Briefly, entorhinal cortex was homogenized in 1X PBS containing protease inhibitor cocktail (1:100, Sigma-Aldrich #P8340) followed by ultracentrifugation at 100,000 g for 1 h at 4 °C. The water-soluble fraction was quantified via the BCA method and 25 μg total protein was blotted onto prewet nitrocellulose membrane in the dot-blot apparatus (Bio-Rad Bio-Dot Microfiltration Apparatus). The membrane was then washed once in TBS and was blocked in 5% nonfat milk in 1X TBS + 0.01% Tween 20 (TBST) for 2 h at room temperature and incubated overnight at 4 °C in polyclonal rabbit anti-amyloid oligomer (A11; 1:5000; EMD Millipore #AB9234). The membrane was then washed three times in TBST and then incubated for 1 h at room temperature in IRDye 800CW donkey anti-rabbit IgG (1:20,000; Li-Cor #925–32213). The membrane was imaged using an Odyssey Fc (800 channel, 30 s acquisition) and protein expression levels were quantified using Image Studio Lite (version 5.2.5; Li-Cor).

### Western blotting

Protein extraction of brain tissue was performed as previously [30]. Antibodies used for Western blot included mouse anti-actin (1:5000, Millipore, Temecula, CA), rabbit anti-amyloid precursor protein (APP, Abcam, Cambridge, MA), mouse anti-phospho-PHF-tau (AT8, 1:500, ThermoFisher Scientific) and mouse anti-tau (tau-5, 1:1000, Millipore, Temecula, CA). The following secondary antibodies were used, mouse anti-HRP (1:10,000, Thermo Scientific, Rockford, Il) and rabbit anti-HRP (1:15,000, Promega, Madison, WI). Protein expression was measured in ImageJ and was normalized to actin.

### Statistical analysis

Stereological quantification was analyzed using two-tail or one-tail (Fig. 5), unpaired *t*-test, or Welch’s unequal variance *t*-test where appropriate. Contextual fear conditioning, pattern separation, and discrimination ratios were analyzed using repeated measures, two-way ANOVA with Holm-Sidak multiple comparison testing. Western blot data was analyzed using two-tail, unpaired *t*-test, or Welch’s unequal variance *t*-test where appropriate. All statistical analysis was done in GraphPad Prism (Version 7.01; GraphPad Software Inc., La Jolla, CA, USA). All data shown represent mean ± S.E.M. and a probability of less than 0.05 was considered statistically significant.

## Results

### Ablation of adult neurogenesis in a FAD mouse model

**Fig. 1:**
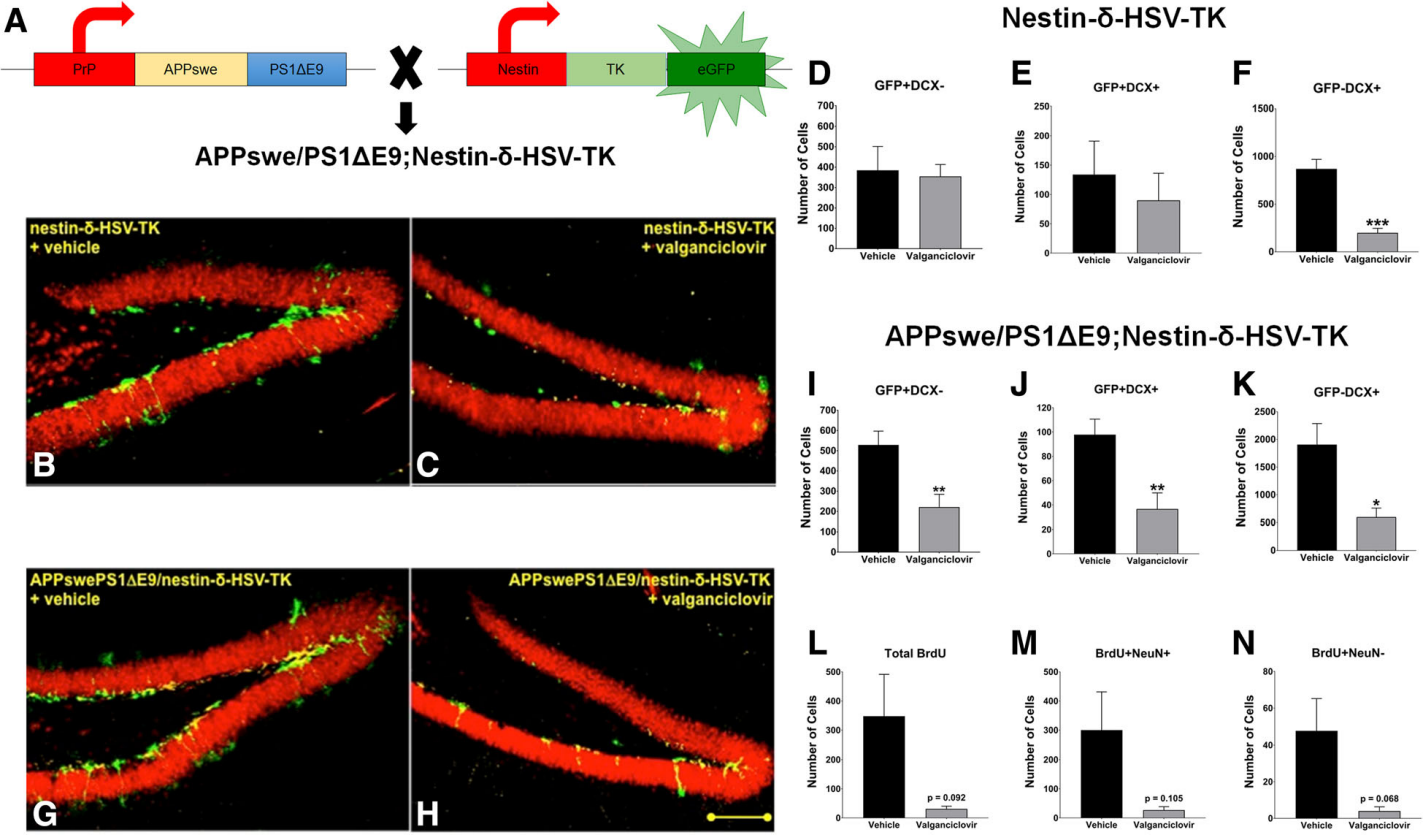
Depletion of hippocampal neurogenesis following treatment of Nestin-δ-HSV-TK and APPswePS1ΔE9/Nestin-δ-HSV-TK mice with valganciclovir. **a** Breeding scheme of APPswe/PS1ΔE9 and Nestin-δ-HSV-TK transgenic mice, to produce transgenic FAD animals that can exhibit selectively ablated neurogenesis, i.e. APPswePS1ΔE9/Nestin-δ-HSV-TK mice. **b**, **c** Confocal images of hippocampal sections of Nestin-δ-HSV-TK transgenic mice stained with anti-NeuN and anti-green fluorescent protein (GFP) + antibodies. Images show less GFP+ neural stem and progenitor cells in the subgranular layer of valganciclovir-treated Nestin-δ-HSV-TK mice (**c**) compared to vehicle-treated Nestin-δ-HSV-TK transgenic mice (**b**). **d**-**f** Quantification of hippocampal neurogenesis in Nestin-δ-HSV-TK that either received vehicle (black bars) or valganciclovir chow (gray bars). There was no change in the number of GFP + DCX- (**d**) or GFP + DCX+ cells (**e**), but there was a significant decrease in the number of GFP-DCX+ cells [(**f**) two-tailed, unpaired *t*-test; *t*_*7*_ = 5.589, *P* = 0.0008]. **g**, **h** Hippocampal sections of 4–5 month old APPswe/PS1ΔE9;Nestin-δ-HSV-TK fed with either vehicle (**g**) or valganciclovir (**h**) chow. Valganciclovir treated animals showed a significant decrease in GFP+ (nestin) and DCX+ expressing cells. **i-n** Quantification of hippocampal neurogenesis in APPswePS1ΔE9/Nestin-δ-HSV-TK that received vehicle- (black bar) or valganciclovir- chow (gray bar). Decrease in total number of (**i**) GFP + DCX- expressing neural progenitor cells (two-tailed, unpaired *t*-test; *t*_*9*_ = 3.253, *P* = 0.0099), (**j**) GFP + DCX+ neuroblasts (two-tailed, unpaired *t*-test; *t*_*9*_ = 3.305, *P* = 0.0092) and (**k**) GFP-DCX+ immature neurons (two-tailed, unpaired *t*-test; *t*_*9*_ = 2.932, *P* = 0.0167) in the subgranular layer (valganciclovir *N* = 5, vehicle *N* = 6). Counts in the granular cell layer (valganciclovir *N* = 4, vehicle N = 5) show decrease in number of (**l**) total BrdU (two-tailed, Welch’s unequal variance *t*-test; *t*_*4.034*_ = 2.204, *P* = 0.0917), (**m**) BrdU + NeuN+ (two-tailed, Welch’s unequal variance *t*-test; *t*_*4.062*_ = 2.077, *P* = 0.1054) and (**n**) BrdU + NeuN- (two-tailed, Welch’s unequal variance *t*-test; *t*_*4.137*_ = 2.457, *P* = 0.0679). Scale bar = 100 μm. *P < 0.05; **P < 0.01

To address the hypothesis that decreased adult neurogenesis will exacerbate the cognitive deficits associated with Alzheimer’s disease, we temporally ablated neural progenitor cells from the brains of FAD-linked APPswe/PS1ΔE9 transgenic mice. For this purpose, we bred the APPswe/PS1ΔE9 mice with the Nestin-δ-HSV-TK transgenic mouse line, [35]. The Nestin-δ-HSV-TK transgenic line contains a modified version of the herpes simplex virus thymidine kinase (TK), as well as an enhanced green fluorescent protein (GFP), driven by the nestin promoter and its second intron regulatory element (Fig. 1a). Administration of valganciclovir in mouse chow, which is specifically phosphorylated by Nestin-δ-HSV-TK, kills dividing nestin expressing cells in these mice by acting as a toxic thymidine analog. The number of GFP-expressing nestin positive cells in the subgranular layer of the hippocampus of Nestin-δ-HSV-TK treated with valganciclovir is reduced compared to vehicle-treated Nestin-δ-HSV-TK mice (Fig. 1b-f). Likewise, a dramatic decrease in GFP+ cells was observed in brain sections of APPswe/PS1ΔE9;Nestin-δ-HSV-TK treated with valganciclovir, compared to vehicle-treated APPswe/PS1ΔE9;Nestin-δ-HSV-TK mice (Fig. 1g-n). To determine the nature of neurogenic deficits in valganciclovir-treated Nestin-δ-HSV-TK and APPswe/PS1ΔE9;Nestin-δ-HSV-TK, we quantified the number of GFP+ neural progenitor cells (NPCs) by unbiased stereology following a two-month valganciclovir treatment. No change in the number of GFP + DCX- (nestin expressing NPCs; Fig. 1d) or GFP + DCX+ (neuroblasts; Fig. 1e) was observed between valganciclovir- and vehicle-treated Nestin-δ-HSV-TK mice. However, a significant reduction in the number of GFP-DCX+ (immature neurons) was observed in Nestin-δ-HSV-TK mice treated with valganciclovir (Fig. 1f; two-tailed, unpaired *t*-test; *t*_*7*_ = 5.589, *P* = 0.0008). Taken together, this may suggest that in the tested conditions, reduced neurogenesis in the valganciclovir treated Nestin-δ-HSV-TK mice is manifested at the immature neuron stage. Interestingly, APPswe/PS1ΔE9;Nestin-δ-HSV-TK mice treated with valganciclovir displayed a significant decrease at earlier stages. Specifically, a significant reduction in total number of nestin expressing NPCs (GFP + DCX-, Fig. 1i; two-tailed, unpaired *t*-test; *t*_*9*_ = 3.253, *P* = 0.0099), neuroblasts (GFP + DCX+, Fig. 1j, two-tailed, unpaired t-test; *t*_*9*_ = 3.305, *P* = 0.0092) and immature neurons (GFP-DCX+, Fig. 1k, two-tailed, unpaired *t*-test; *t*_*9*_ = 2.932, *P* = 0.0167) in the subgranular layer following treatment (Valganciclovir *N* = 5, Vehicle *N* = 6). This may suggest that in the APPswe/PS1ΔE9;Nestin-δ-HSV-TK mice, treatment with valganciclovir affects earlier neurogenic populations compared to the Nestin-δ-HSV-TK mice and is manifested by a significant reduction in the number of NPCs, neuroblasts and immature neurons. This may further support previous reports suggesting impairment of NPCs in FAD [30, 38]. To assess the impact on the number of newly born neurons we quantified the number of BrdU + NeuN+ cells in the granular cell layer of the DG of the APPswe/PS1ΔE9;Nestin-δ-HSV-TK mice. Unbiased stereology demonstrated a trending decrease in the total number of BrdU+ (Fig. 1l, two-tailed, Welch’s unequal variance *t*-test; *t*_*4.034*_ = 2.204, *P* = 0.0917), BrdU + NeuN+ new neurons (Fig. 1m, two-tailed, Welch’s unequal variance *t*-test; *t*_*4.062*_ = 2.077, *P* = 0.1054), as well as in the number of new glia BrdU + NeuN- cells (Fig. 1n, two-tailed, Welch’s unequal variance t-test; *t*_*4.137*_ = 2.457, *P* = 0.0679) following valganciclovir treatment (Valganciclovir *N* = 4, Vehicle *N* = 5). Combined, this data demonstrates a successful ablation of adult neurogenesis in APPswe/PS1ΔE9;Nestin-δ-HSV-TK animals, resulting in less new neurons and glia in the granular cell layer of the DG. In addition, these results may imply that NPCs and neuroblasts are more vulnerable in the brains of APPswe/PS1ΔE9;Nestin-δ-HSV-TK mice compared to the Nestin-δ-HSV-TK mice.

### Ablation of adult neurogenesis in FAD mice induces deficits in the contextual fear conditioning task

**Fig. 2:**
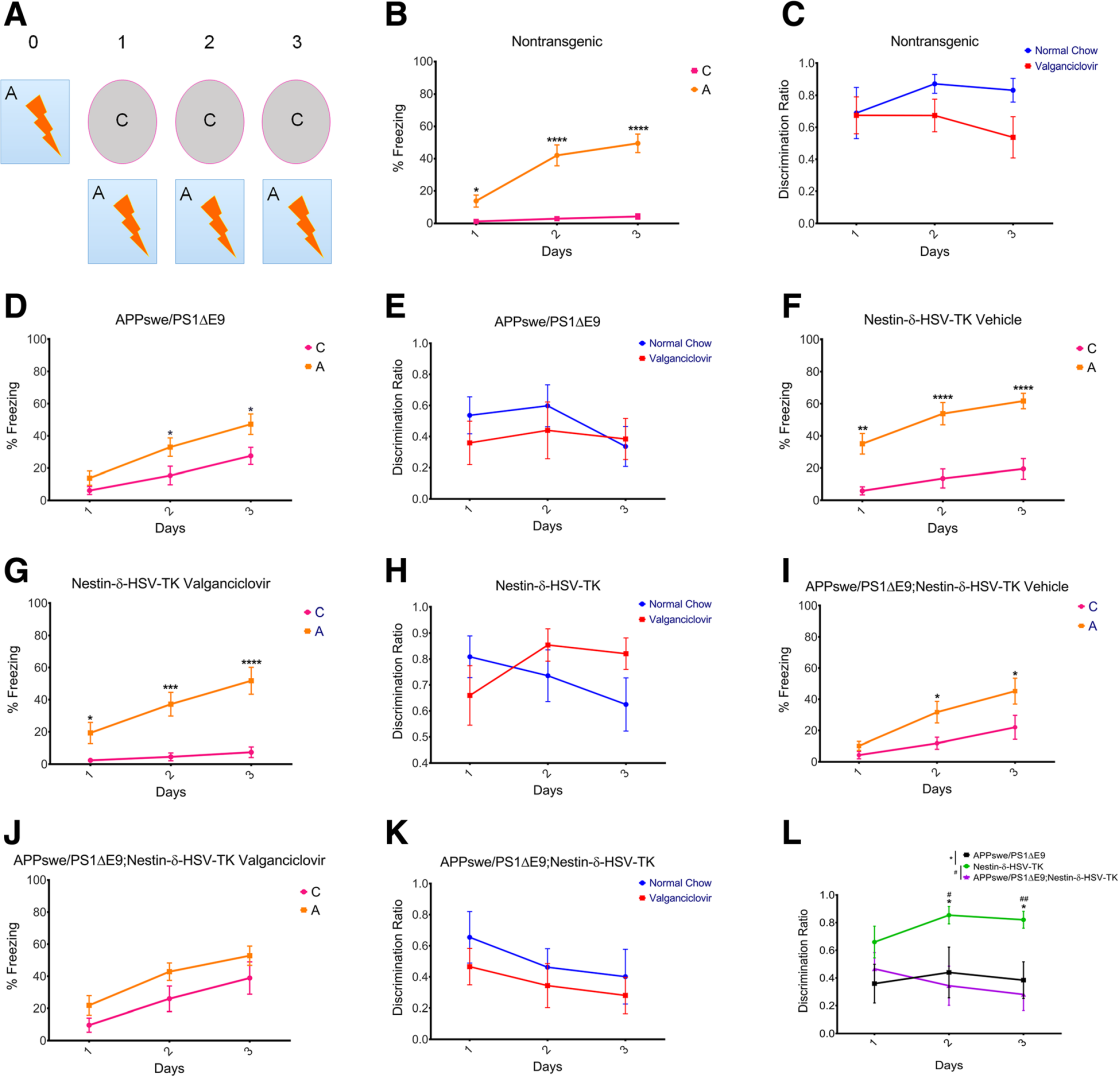
Depletion in neurogenesis enhances impairments in contextual fear conditioning. **a** Schematic presentation of the experimental paradigm used. A (shock) and C (no shock) are distinct contexts. Mice were fed with either vehicle or valganciclovir chow after weaning for 1.5 months prior to the contextual fear conditioning task. **b**, **d**, **f**, **g** Based on anticipatory freezing behavior nontransgenic (*N* = 11) **b**), APPswePS1ΔE9 (*N* = 14) (**d**), vehicle-fed Nestin-δ-HSV-TK [((**f**), *N* = 12)] and valganciclovir-fed Nestin-δ-HSV-TK [(**g**), (*N* = 8)] mice are able to distinguish context A from C (repeated measures two-way ANOVA, **b**. Context: F_1,20_ = 46.64, *P* < 0.0001; **d**. Context: F_1,26_ = 6.36, *P* = 0.0182; **f**. Context: F_1,22_ = 31.40, *P* < 0.0001; **g**. Context: F_1,14_ = 19.72, *P* = 0.0006). **i** vehicle- treated APPswe/PS1ΔE9;Nestin-δ-HSV-TK (*N* = 7) are able to distinguish between the contexts (repeated measures two-way ANOVA, **i**. Context: F_1,12_ = 7.90, *P* = 0.0157). **j** valganciclovir-treated APPswe/PS1ΔE9;Nestin-δ-HSV-TK (*N* = 9) mice are unable to distinguish between the two contexts (repeated measures two-way ANOVA, **j**. Context: F_1,16_ = 2.82, *P* = 0.1125) .**c**, **e**, **h**, **k** Discrimination ratios in (**c**) nontransgenic, (**e**) APPswe/PS1ΔE9, (**h**) Nestin-δ-HSV-TK, and (**k**) APPswe/PS1ΔE9;Nestin-δ-HSV-TK revealed no significant differences in the animal’s ability to discriminate between contexts between days; however, (**h**) Nestin-δ-HSV-TK mice had a significant Context x Days interaction (repeated measures two-way ANOVA, Context x Days: F_2,36_ = 3.56 *P* = 0.0388). **l** Valganciclovir-fed Nestin-δ-HSV-TK exhibited significantly better abilities to discriminate between contexts A and C compared to valganciclovir-fed APPswe/PS1ΔE9 and APPswe/PS1ΔE9;Nestin-δ-HSV-TK mice (repeated measures two-way ANOVA, *Nestin-δ-HSV-TK vs. APPswe/PS1ΔE9;, ^#^Nestin-δ-HSV-TK vs. APPswe/PS1ΔE9;Nestin-δ-HSV-TK) with the genotype having a significant influence on discrimination between contexts A and C (repeated measures two-way ANOVA, Genotype: F_2,22_ = 7.00 *P* = 0.0044). *P < 0.05; **P < 0.01; ***P < 0.001; ****P < 0.0001; ^#^ *P* < 0.05; ^##^ *P* < 0.01

To determine cognitive performance of FAD mice following depletion of neurogenesis, mice were tested in the contextual fear conditioning and the DG-specific pattern separation tasks as previously described [36, 39]. In the contextual fear-conditioning task (Fig. 2a), age matched wild type (Nontransgenic, *N* = 11, Fig. 2b) and APPswe/PS1ΔE9 (Fig. 2d, *n*=14) mice that were fed with vehicle chow, were able to successfully distinguish between the two contexts, based on percentage freezing, by the end of the second day. Discrimination ratio shows no difference between vehicle- and valganciclovir-fed wild type (Fig. 2c) or APPswe/PS1ΔE9 mice (Fig. 2d), suggesting no side effect of the valganciclovir on animals’ behavior. Similarly, Nestin-δ-HSV-TK animals fed with either vehicle- or valganciclovir-containing chow were also able to successfully learn the task (vehicle-treated Nestin-δ-HSV-TK *N* = 12, valganciclovir- treated Nestin-δ-HSV-TK *N* = 8, Fig. 2f-h respectively), suggesting that ablation of adult neurogenesis alone is insufficient for the disruption of this behavioral task. Vehicle treated APPswe/PS1ΔE9;Nestin-δ-HSV-TK animals exhibited some difficulty learning the task on the first day, but successfully distinguished between the two contexts on day 2 and 3 (*N* = 7, Fig. 2i). Importantly, valganciclovir-fed APPswe/PS1ΔE9;Nestin-δ-HSV-TK mice were unable to distinguish between context A or C on any of the days of the test (*N* = 9, Fig. 2j). It is important to note that this deficit was only observed with the combination of ablated neurogenesis and the FAD mouse background. Nevertheless, discrimination ratios revealed no significant difference in the animal’s ability to discriminate between contexts between days (Fig. 2k), which may suggest that the effect is very mild. However, valganciclovir-fed Nestin-δ-HSV-TK mice had a significant Context x Days interaction (Fig. 2l, repeated measures two-way ANOVA, Context x Days: F_2,36_ = 3.56 *P* = 0.0388). Furthermore, on Days 2 and 3 valganciclovir-fed Nestin-δ-HSV-TK exhibited significantly increased abilities to discriminate between contexts A and C compared to valganciclovir-fed APPswe/PS1ΔE9;Nestin-δ-HSV-TK and APPswe/PS1ΔE9 mice (Fig. 2l; repeated measures two-way ANOVA, * *P* < 0.05, ^#^ *P* < 0.05, ^##^ *P* < 0.01) with the genotype having a significant influence on discrimination between contexts A and C (repeated measures two-way ANOVA, Genotype: F_2,22_ = 7.00 *P* = 0.0044). To examine whether freezing behavior is the result of anxiety rather than learning, mice were subjected to the Light/Dark box anxiety task. However we found no differences in anxiety level between any of the groups (data not shown), suggesting that these observed deficits are not a result of increased anxiety in the APPswe/PS1ΔE9;Nestin-δ-HSV-TK animals.

### Ablation of adult neurogenesis in FAD mice induces deficits in the pattern separation task

**Fig. 3:**
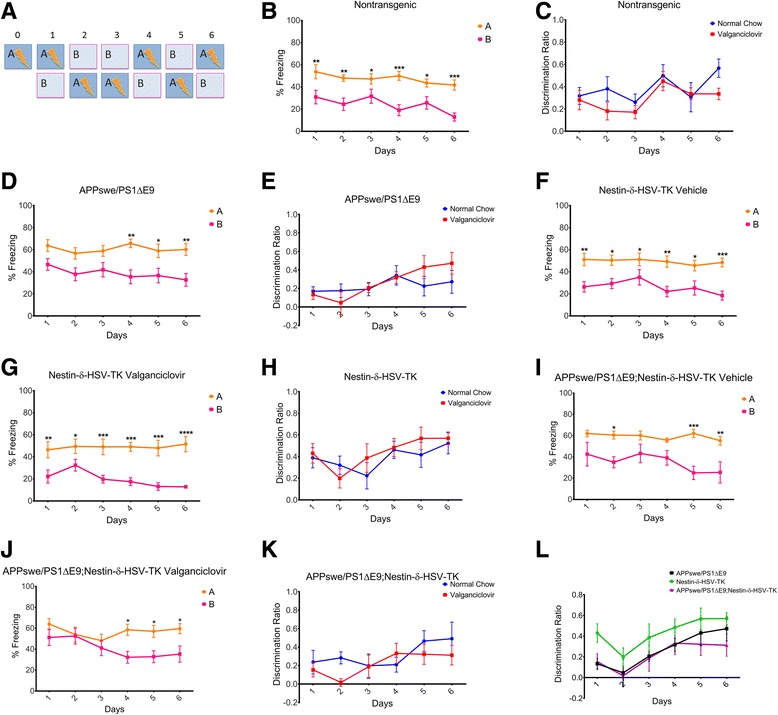
Ablation of neurogenesis compromises pattern separation in APPswe/PS1ΔE9;Nestin-δ-HSV-TK mice. **a** Schematic presentation of the experimental paradigm used to test pattern separation. Two very similar contexts, A (shock) and B (no shock). Nontransgenic [*N* = 11, (**b**)], APPswe/PS1ΔE9 [*N* = 13, (**d**)], vehicle- treated Nestin-δ-HSV-TK [*N* = 12, (**f**)**]** and valganciclovir- treated Nestin-δ-HSV-TK [*N* = 7, (**g**)] mice are able to distinguish context A from B (repeated measures two-way ANOVA, **b**. Context: F_1,20_ = 18.20, *P* = 0.0004; **d**. Context: F_1,16_ = 7.06, *P* = 0.0172; **f**. Context: F_1,22_ = 13.24, *P* = 0.0014; **g**. Context: F_1,12_ = 22.87, *P* = 0.0004). **i** vehicle-treated APPswe/PS1ΔE9;Nestin-δ-HSV-TK mice (*N* = 7) exhibit similar but not identical learning patterns to APPswe/PS1ΔE9 mice (repeated measures, two-way ANOVA, F_1,10_ = 18.09, *P* = 0.0017). **j** Valganciclovir-treated APPswe/PS1ΔE9;Nestin-δ-HSV-TK (*N* = 8) mice are unable to distinguish between the two contexts for the first three days (repeated measures, two-way ANOVA, F_1,14_ = 4.45, *P* = 0.0534). **c**, **e**, **h**, **k** Discrimination ratios in (**c**) nontransgenic, (**e**) APPswe/PS1ΔE9, (**h**) Nestin-δ-HSV-TK, and (**k**) APPswe/PS1ΔE9;Nestin-δ-HSV-TK revealed no significant difference in the animal’s ability to discriminate between contexts between days. **l** There were no significant differences in discrimination ratios between days for valganciclovir-fed Nestin-δ-HSV-TK, APPswe/PS1ΔE9, and APPswe/PS1ΔE9;Nestin-δ-HSV-TK mice but there was a significant effect overall across days (repeated measures two-way ANOVA, Days: F_5,100_ = 10.50 *P* < 0.0001). *P < 0.05; **P < 0.01; ***P < 0.001; ****P < 0.0001

We next asked whether depletion of neurogenesis in the FAD mice would induce difficulty learning in a known DG-specific task, pattern separation. The challenge in this task is the high similarity between the two contexts, and typically the learning process is longer compared to the contextual fear conditioning (Fig. 3a).

Vehicle chow-fed age matched wild type (Nontransgenic, *N* = 11, Fig. 3b) or APPswe/PS1ΔE9 animals (*N* = 13, Fig. 3d) were able to successfully distinguish between the two contexts, based on percentage freezing, by the end of the pattern separation task (repeated measures two-way ANOVA, ** *P* < 0.01, ***P < 0.001). Discrimination ratios of vehicle and valganciclovir-treated wild type (Fig. 3c) and APPswe/PS1ΔE9 (Fig. 3e) show no difference between the treatment groups, excluding the possibility of side effect of valganciclovir on mouse behavior. Unexpectedly, Nestin-δ-HSV-TK animals fed with either vehicle or valganciclovir containing chow were able to successfully learn the task (Vehicle-treated *N* = 12, valganciclovir-treated *N* = 7, Fig. 3f-h; repeated measures two-way ANOVA, * *P* < 0.05, **P < 0.05, ***P < 0.001, ****P < 0.0001), suggesting that either ablation of adult neurogenesis using the Nestin-δ-HSV-TK transgenic line is insufficient for the disruption of this behavioral task, or that our specific contexts were not sufficiently challenging, masking the deficit. Interestingly, vehicle-fed APPswe/PS1ΔE9;Nestin-δ-HSV-TK were able to discriminate between the tasks albeit not all days were statistically significant (*N* = 7, Fig. 3i; repeated measures two-way ANOVA, * *P* < 0.05, **P < 0.05, ***P < 0.001). This may suggest a genetic background effect in comparison to vehicle-fed APPswe/PS1ΔE9 mice. Importantly, ablation of neurogenesis in the APPswe/PS1ΔE9;Nestin-δ-HSV-TK mice completely disrupted the performance of mice in the first three days of the task (*N* = 8, Fig. 3j; repeated measures two-way ANOVA, * *P* < 0.05). Discrimination ratios within genotype revealed no significant differences in the animal’s ability to discriminate between contexts between days (Fig. 3k), suggesting a mild behavioral effect of neurogenesis in this task. Additionally, when comparing discrimination ratios between valganciclovir-fed Nestin-δ-HSV-TK, APPswe/PS1ΔE9 and APPswe/PS1ΔE9;Nestin-δ-HSV-TK mice there was no significance within days but there was an overall days effect among the groups (Fig. 3l; repeated measures two-way ANOVA, Days: F_5,100_ = 10.50 *P* < 0.0001).

### Ablation of adult neurogenesis enhances tau hyperphosphorylation in FAD mice

**Fig. 4:**
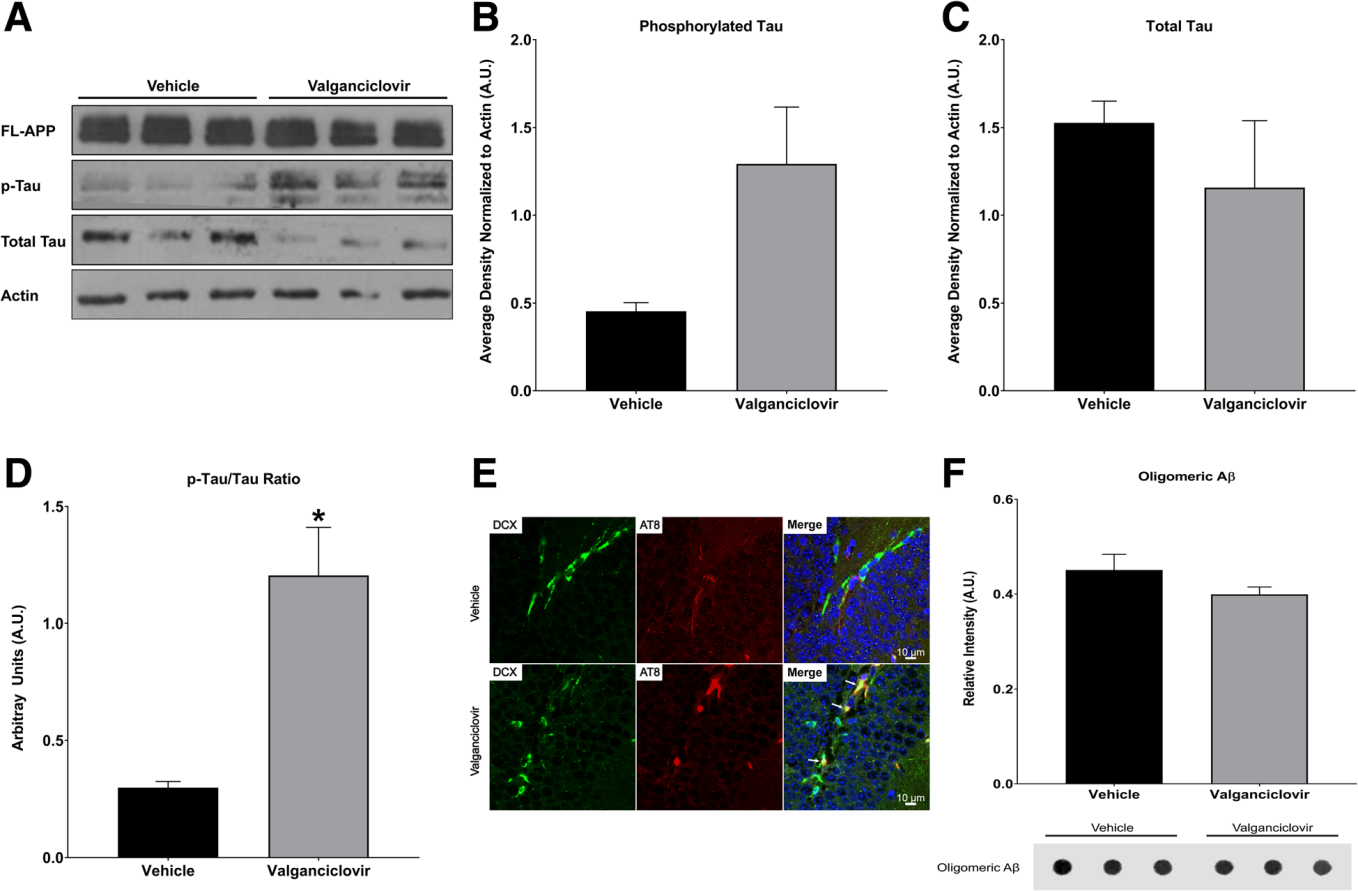
Ablation of neurogenesis increases the expression of phosphorylated tau in the hippocampus of APPswe/PS1ΔE9;Nestin-δ-HSV-TK mice. **a** Western blot of hippocampal protein extract from 4 month old APPswe/PS1ΔE9;Nestin-δ-HSV-TK mice fed with vehicle or valganciclovir chow for 3 months (*N* = 3) shows that the expression level of full length amyloid precursor protein (APP) is comparable between the groups. However, there was a decrease in level of total tau and an increase in level of AT8-positive hyperphosphorylated tau (p-tau) in the valganciclovir-treated APPswe/PS1ΔE9;Nestin-δ-HSV-TK mice. **b**-**d** Semi-quantitative analysis of Western blot using Image J normalized to actin. There was a trending increase in levels of (**b**) p-tau (AT8; two-tail, Welch’s unequal variance *t*-test, *t*_*2.094*_ = 2.553, *P* = 0.1197), with no change in (**c**) total tau (Tau-5, two-tail, unpaired t-test, *t*_*4*_ = 0.9195, *P* = 0.4099) and (**d**) a significant increase in the ratio of p-tau to total tau (two-tailed, unpaired *t*-test; *t*_*4*_ = 4.368, *P* = 0.0120) in the brains of valganciclovir-treated APPswe/PS1ΔE9;Nestin-δ-HSV-TK. *P < 0.05. **e** Doublecortin (DCX) positive neuroblasts and immature neurons in the dentate gyrus of APPswe/PS1ΔE9;Nestin-δ-HSV-TK mice fed with valganciclovir chow are immunoreactive for the anti-p-tau antibodies AT8. Immunoreactivity was not detected in APPswe/PS1ΔE9;Nestin-δ-HSV-TK mice fed with vehicle. Scale bar = 10 μm. **f** Dot blot analysis of water soluble protein extract of the entorhinal cortex of APPswe/PS1ΔE9;Nestin-δ-HSV-TK mice fed with valganciclovir or normal chow reveals no difference in the level of A11 immunoreactive oligomeric Aβ between the groups (*N* = 3)

Next, we asked whether, in addition to learning and memory, hippocampal neurogenesis would affect AD neuropathology. Western blot analysis of hippocampal protein lysate of vehicle- and valganciclovir-treated APPswe/PS1ΔE9;Nestin-δ-HSV-TK animals showed that the levels of phosphorylated tau, as detected by AT8 antibodies, demonstrated a trending increase following ablation of adult neurogenesis in the valganciclovir-treated APPswe/PS1ΔE9;Nestin-δ-HSV-TK (Fig. 4a,b). Total tau expression levels did not change (tau-5, Fig. 4a,c), while the ratio of phosphorylated tau to total tau was significantly increased (Fig. 4d; two-tailed, unpaired *t*-test; *t*_*4*_ = 4.368, *P* = 0.0120). This observation suggests that ablation of adult neurogenesis induces upregulation of tau hyperphosphorylation in the hippocampus. Immunohistochemical analysis of hippocampal sections stained for phosphorylated tau (p-tau) and doublecortin (DCX) clearly show AT8 immunoreactivity in DCX+ neuroblasts and new neurons in valganciclovir-treated APPswe/PS1ΔE9;Nestin-δ-HSV-TK, but not in vehicle-treated APPswe/PS1ΔE9;Nestin-δ-HSV-TK mice (Fig. 4e). This may suggest that depletion of neurogenesis compromises the regulation of tau phosphorylation in newly differentiated neurons. Given the critical role of tau in neuronal maturation, this may suggest that the reduced level of neurogenesis may lead to the incorporation of aberrant new neurons into the hippocampus. To make sure that the observed alterations in tau phosphorylation are not a side effect of the valganciclovir treatment, we examined p-tau expression in vehicle- and valganciclovir- treated wild type mice and observed no change in p-tau expression (Additional file 1: Figure S1). This suggests that induction of hyperphosphorylated tau observed in the valganciclovir-treated APPswe/PS1ΔE9;Nestin-δ-HSV-TK was due to the depletion of neurogenesis.

To address the effect of neurogenesis on the amyloidogenic pathway we examined levels of full length APP in hippocampal protein lysates of vehicle- and valganciclovir-treated APPswe/PS1ΔE9;Nestin-δ-HSV-TK animals. We observed that APP levels do not change after ablation of neurogenesis (*N* = 3, Fig. 4a). In addition, the level of oligomeric Aβ was comparable in water-soluble protein extracts prepared from the entorhinal cortices of these mice (Fig. 4f). These results suggest that the effect of depletion of neurogenesis on tau may not be modulated by amyloidosis.

### Ablation of adult neurogenesis modifies hippocampal circuit activity

**Fig. 5:**
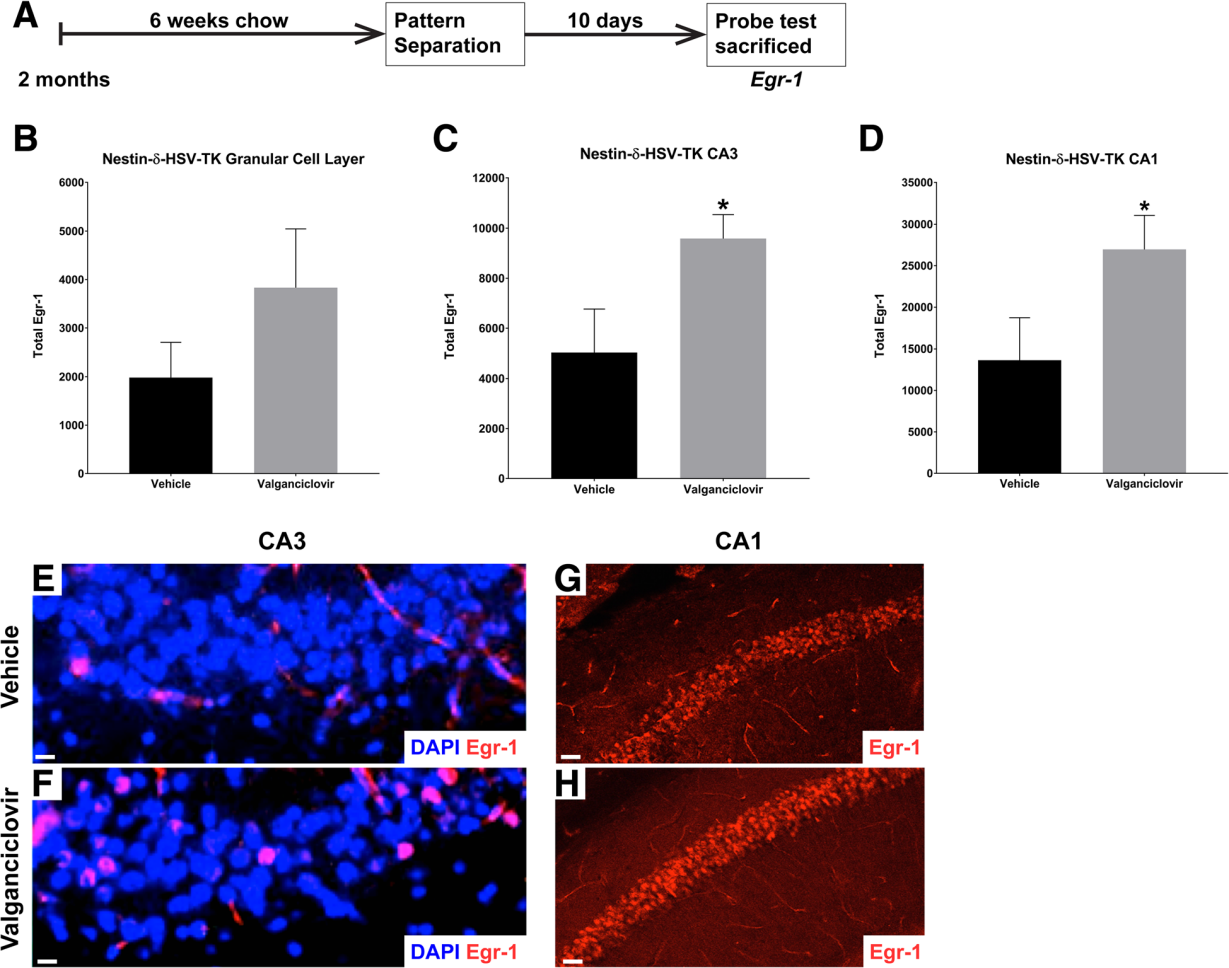
Over-activation of hippocampal neurons following long term memory retrieval in neurogenesis-depleted Nestin-δ-HSV-TK mice (**a**) Experimental design. Nestin-δ-HSV-TK mice (*N* = 4) were fed with vehicle or valganciclovir chow following weaning for six weeks before performing pattern separation testing. Ten days after behavioral testing, a single probe test, where the mouse was placed only in the shock context A, was performed. Then, the animals were sacrificed for analysis of changes in Egr-1 (*zif268*) expression in the hippocampal circuitry. **b**-**d** Quantification of Egr-1 positive cells in the hippocampus. The total number of Egr-1 positive cells showed a trending increase in the granular cell layer of the dentate gyrus (**b**), with significant increases in the CA3 region (**c**) (one-tailed, unpaired *t*-test; *t*_*4*_ = 2.302, *P* = 0.0414), and the CA1 region (**d**) (one-tailed, unpaired *t*-test; *t*_*6*_ = 2.039, *P* = 0.0438). **e**-**h** Images of DAPI + Egr-1+ cells in the CA3 (**e**, **f**) and Egr-1+ cells in the CA1 (**g**, **h**) regions of vehicle- or valganciclovir-treated Nestin-δ-HSV-TK mice reveal more Egr-1+ cells in the valganciclovir-treated Nestin-δ-HSV-TK mice. Scale bar = 15 μM (E,F), 75 μM (**g**, **h**)

To assess the impact of loss of neurogenesis on the activity of the hippocampal circuitry we examined expression of the immediate early gene Egr-1 (zif268), as an indicator of neuronal activation following learning. For this purpose, Nestin-δ-HSV-TK mice fed with vehicle or valganciclovir were subject to pattern separation, as above. To assess the neuronal population activated by the long-term memory of context A, ten days after task completion, a single probe trial of only the shock context was performed, and mice were immediately sacrificed and examined for Egr-1 expression (Fig. 5a). Using unbiased stereology the number of Egr-1 expressing cells was quantified in the granular cell layer of the DG, the CA1 region, and the CA3 region of these mice (*N* = 4). Ablation of adult neurogenesis caused a trending increase in the number of Egr-1 expressing cells in the granular cell layer of the DG (Fig. 5b), with a significant increase in the CA3 region (Fig. 5c,e,f; one-tailed, unpaired *t*-test; *t*_*4*_ = 2.302, *P* = 0.0414), and the CA1 region (Fig. 5d,g,h; one-tailed, unpaired *t*-test; *t*_*6*_ = 2.039, *P* = 0.0438). These results suggest that eliminating the presence of new neurons relieves inhibitory circuitry in the DG-CA3-CA1. Alternatively, this may be the result of decreased excitatory synapses onto interneurons in the hilus.

## Discussion

This study addresses three fundamental questions. First, we examined the role of neurogenesis in cognitive deficits in AD. We show that depletion of neurogenesis induces mild learning and memory impairments in APPswe/PS1ΔE9 mice. Previous reports show that in the APPswe/PS1ΔE9 mouse model cognitive deficits can develop as early as six months, including impairment in the fear-conditioning task [40]. We show that depletion of neurogenesis in the brains of APPswe/PS1ΔE9 mice exacerbates performance deficits in this task inducing deficits at 4 months of age. It should be noted that APPswe/PS1ΔE9 mice exhibit impairments in neurogenesis as early as 2 months of age characterized by both reduction in neurogenesis as well as defective neuronal differentiation [33]. Thus, the comparison between vehicle- and valganciclovir-treated APPswe/PS1ΔE9; Nestin-δ-HSV-TK mice is between defective and ablated neurogenesis, respectively. That said, in contrast to vehicle-treated, valganciclovir-treated APPswe/PS1ΔE9; Nestin-δ-HSV-TK mice were unable to learn the contextual fear conditioning task at all. In the case of pattern separation, valganciclovir-treated APPswe/PS1ΔE9; Nestin-δ-HSV-TK mice were unable to learn the task for the first three days. These results are the first evidence that learning and memory impairments in AD can be induced by compromising neurogenesis. It should be noted that mice were subjected to pattern separation following contextual fear conditioning. Thus, we cannot exclude the possibility that the former played a role in training them better for the latter. In such case, this may mask the true level of the learning deficits of the valganciclovir-treated APPswe/PS1ΔE9; Nestin-δ-HSV-TK mice, who without training would likely have taken longer to learn the task.

Notably, Valganciclovir-treated Nestin-δ-HSV-TK mice, not on an AD background, did not exhibit deficits in either contextual conditioning or pattern separation. While neurogenesis has not been directly implicated in contextual fear conditioning, its role in pattern separation is well established [39, 41]. This may suggest that either ablation of adult neurogenesis using the Nestin-δ-HSV-TK transgenic line is not sufficient to disrupt learning and memory, or that our specific contexts were not sufficiently challenging to unravel deficits. While previous lesion studies of the hippocampus have shown deficits in pattern separation, more specific genetic modulation of adult neurogenesis demonstrates mixed results [1]. For example, [42] found deficits in pattern separation after ablation of nestin expressing cells using the Nestin-rtTA, TRE-BAX transgenic line, while [43] ablated proliferating GFAP expressing cells in the hippocampus and observed no deficits in pattern separation [42, 43]. Further studies will need to be done to determine if our result is due to the specific transgenic line used or if aspects of our study such as duration of feeding with valganciclovir chow, age/gender of the animals or specific aspects of our contextual paradigm are underlying this difference.

Second, we addressed the role of neurogenesis in modulation of tau pathology. We show that ablation of neurogenesis upregulates levels of hyperphosphorylated tau, and that this effect is neurogenesis- rather than valganciclovir-dependent. This may strongly suggest that neurogenesis plays a major role in hippocampal maintenance, and that depletion of neurogenesis compromises neuronal viability. In addition, induction of hyperphosphorylated tau in neural progenitor cells suggests that depletion of neurogenesis may compromise the integrity and functionality of new neurons in the hippocampus. In contrast, we did not detect any effect of neurogenesis on the level of full length APP or oligomeric Aβ. Our results are in agreement with studies reporting lack of direct association between amyloidosis and neurogenesis. For instance, previous studies suggest that enhanced neurogenesis does not alter amyloid plaque load [44]. In turn, amyloid does not play a major role in impairments of neurogenesis in FAD [45].

Third, we addressed the role of neurogenesis in regulation of the hippocampal circuit. It is thought that the DG regulates pattern separation either by encoding similar inputs with differential firing rates of place cells (rate remapping) or through the recruitment of non-overlapping engrams (global remapping) [46, 47]. However, the exact mechanism is not fully elucidated. A recent study suggests that neurogenesis modifies the excitability of mature DG neurons without affecting excitability in the CA3 [48]. Another study shows that genetic suppression of adult neurogenesis impairs pattern separation by increasing overlap between engrams of differing contexts in CA3 cells [49]. The latter study, in agreement with ours, examined neuronal activation following a learning task, which enhanced neuronal response in the hippocampus. We show that depletion of neurogenesis in valganciclovir-treated Nestin-δ-HSV-TK mice induces the activation of more neurons in the hippocampus, and that this neuronal over-activation takes place in the DG, CA3 and CA1. This result suggests that following depletion of neurogenesis, more neurons get activated, thus increasing the chance of coding overlap, which would subsequently impair pattern separation. Notably, our results suggest that the effect of ablation of neurogenesis on the hippocampal circuit is broader than previously reported. Our result may also suggest that neurogenesis plays a major role in memory storage and recall by regulating inhibitory networks in the hippocampus. One possibility is that new neurons affect the circuit by modulating the activity of the older mature granular neurons and affecting their feedback inhibition, which in turn, affects the CA3. Alternatively, they may directly affect feedforward excitation and inhibition to the CA3.

In summary, this study implies that depletion of hippocampal neurogenesis, as occurs in the adult brain as a function of age, may compromise hippocampal function and induce learning and memory deficits and some neuronal pathology. Future studies should address the possibility that enhanced neurogenesis may be protective and reduce the risk for AD.

## Conclusions

The results of this study imply that neurogenesis plays an important role in the regulation of inhibitory circuitry of the hippocampus. In addition, this study suggests that deficits in adult neurogenesis may contribute to cognitive impairments, tau hyperphosphorylation in new neurons and compromised hippocampal circuitry in Alzheimer’s disease.

## Additional file

Additional file 1: Figure S1. Valganciclovir treatment does not alter tau phosphorylation. Western blot of hippocampal protein extract from 4 months old nontransgenic mice fed with vehicle or valganciclovir chow for 3 months (*N* = 2). There was no change in the expression of p-tau as recognized by AT8 antibodies in extracts of valganciclovir-versus vehicle-treated mice (TIFF 1521 kb)

## Abbreviations

APPswe/PS1ΔE9: Coexpression of Amyloid Precursor Protein mutated at KM670/671NL (APPswe) and presenilin-1 lacking exon 9 (PS1ΔΕ9).
3xTg-AD: Familial Alzheimer’s disease mouse model carrying three mutant transgenes: APP KM670/671NL (Swedish), MAPT P301L, PSEN1 M146V
AD: Alzheimer’s Disease
APP: Amyloid Precursor Protein
BrdU: 5′-bromo-2′-deoxyuridine
CA1,3: *Cornu Ammonis*, Hippocampal subfield 1,3
CREB: cAMP response element binding protein
DAPI: 4′,6-diamidino-2-phenylindole
DCX: Doublecortin
DG: Dentate Gyrus
FAD: Familial Alzheimer’s Disease
GFP: Green Fluorescent Protein
IgG: Immunoglobulin G
NeuN: Neuronal Nuclear antigen
NPC: Neural Progenitor Cell
PS1: Presenilin 1
p-tau: Phosphorylated tau
sAPPα: Soluble Amyloid Precursor Protein Alpha
TBST: Tris Buffered Saline with 0.01% Tween 20
Zif268 (Egr-1): Zinc Finger Transcription Factor 268
δ-HSV-TK: Modified version of the Herpes Simplex Virus Thymidine Kinase
