# Supplementary figures and images for: Depletion of adult neurogenesis exacerbates cognitive deficits in Alzheimer’s disease by compromising hippocampal inhibition
Source: Mol Neurodegener. 2017 Sep 8;12:64. doi: 10.1186/s13024-017-0207-7 (PMC5591545; doi:10.1186/s13024-017-0207-7)

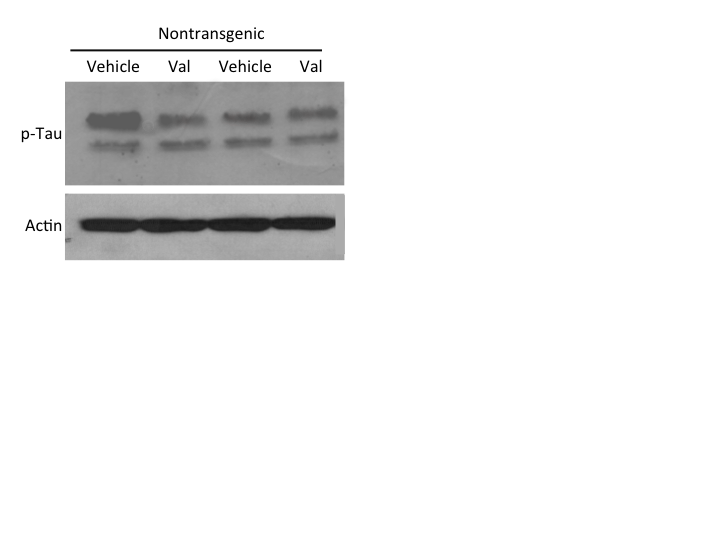

Supplement: Additional file 1: Figure S1. — Valganciclovir treatment does not alter tau phosphorylation. Western blot of hippocampal protein extract from 4 months old nontransgenic mice fed with vehicle or valganciclovir chow for 3 months (N = 2). There was no change in the expression of p-tau as recognized by AT8 antibodies in extracts of valganciclovir-versus vehicle-treated mice (TIFF 1521 kb) [file 13024_2017_207_MOESM1_ESM.tiff]
